# 1001. Chronic Colonization with Toxigenic *Clostridioides difficile* Strains Drives Colonic Tumorigenesis in Mice

**DOI:** 10.1093/ofid/ofab466.1195

**Published:** 2021-12-04

**Authors:** Julia L Drewes, Jie Chen, Reece Knippel, Nicholas Markham, Jada Domingue, June Chan, Madison McMann, Courtney Stevens, Ada J Tam, James White, Fuad Mohammad, Xinqun Wu, Shaoguang Wu, Patricia J Simner, Karen C Carroll, Karen C Carroll, Hua Ding, Martha Shrubsole, Franck Housseau, Ken Lau, Robert Coffey, Cynthia L Sears, Cynthia L Sears

**Affiliations:** 1 Johns Hopkins University School of Medicine, Baltimore, Maryland; 2 Vanderbilt University Medical Center, Nashville, Tennessee; 3 Resphera Biosciences, Baltimore, Maryland; 4 Johns Hopkins School of Medicine, Baltimore, MD; 5 Johns Hopkins University School of Public Health, Baltimore, Maryland; 6 Johns Hopkins University, Baltimore, Maryland; 7 Vanderbilt University, Nashville, Tennessee; 8 VUMC, Nashville, Tennessee; 9 Johns Hopkins, Baltimore, MD

## Abstract

**Background:**

Long-term effects of chronic and/or recurrent *C. difficile* infections (CDI) are not well understood, and any potential role of CDI in colorectal cancer (CRC) risk is presently unknown. While pursuing efforts to identify novel procarcinogenic microbes, we identified two mucosal slurries from CRC patients (3728T and 3752T) that were tumorigenic in germ-free (GF) *Apc*^*Min/+*^ mice. Surprisingly, both of these CRC patient slurries were positive for *C. difficile* by 16S rRNA amplicon sequencing. Given the ability of other chronic infections to promote tumorigenesis (e.g., *H. pylori*), we hypothesized that chronic colonization with *C. difficile* could promote tumorigenesis in the colon.

**Methods:**

A consortium of 30 bacterial isolates including a toxigenic *tcdA*+ *tcdB*+ *C. difficile* strain (CIm_3728T) was cultured from GF *Apc*^*Min/+*^ mice gavaged with the 3728T slurry. This consortium was gavaged into additional GF *Apc*^*Min/+*^ mice with or without *C. difficile* strains CIm_3728T, CIm_3752T (isolated from mice gavaged with the 3752T slurry), or isogenic *tcdA*/*tcdB* mutants of the M7404 R027 strain. Single cell RNA sequencing (scRNAseq), high dimensional (HD) flow cytometry, and fluorescence *in situ* hybridization (FISH) with EUB338 and Cd198 probes were performed on distal colons from mice gavaged with either complex CRC slurries or the 3728T isolates with CIm_3728T.

**Results:**

*C. difficile* strains drove tumorigenesis of the 3728T isolate mixture (**Fig. 1A,B**). Tumorigenesis was associated with early procarcinogenic signaling and spatial changes including induction of Wnt signaling in colonic epithelial progenitor cells by scRNAseq, IL-17 induction in immune cells by HD flow cytometry, and bacterial biofilm invasion deep into epithelial crypts by FISH. Tumorigenesis correlated with chronic colonization with toxigenic strains of *C. difficile* and was toxin-dependent, as toxin mutant strains (M7404 *tcdA-tcdB*-) did not induce tumors.

Figure 1. C. difficile strains from CRC patients induce distal colonic tumorigenesis in germ-free (GF) ApcMin/+ mice.

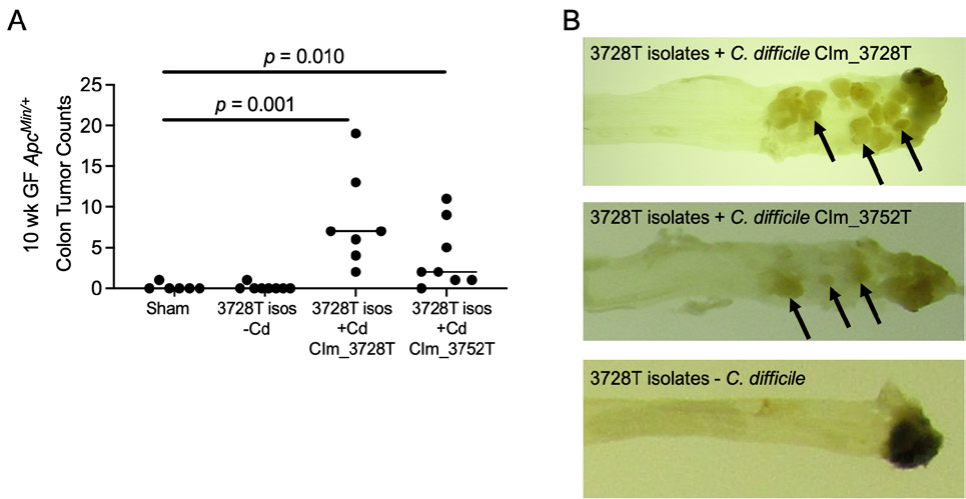

A consortium of 30 bacteria, including C. difficile, were isolated from mice gavaged with the 3728T human CRC mucosal slurry. These isolates were then gavaged into additional GF ApcMin/+ mice, with or without C. difficile isolates from mice gavaged with the 3728T slurry or 3752T slurry. (A) Colonic tumor numbers in GF ApcMin/+ mice at 10 wk p.i. demonstrate that C. difficile (Cd) drives the tumorigenesis of this 30-member bacterial consortium. (B) Gross tumors can be observed in the colon of a representative mouse gavaged with the 3728T isolates with the CIm_3728T (top) or CIm_3752T (middle) strain of C. difficile but not in a mouse gavaged with the isolates lacking C. difficile (bottom).

**Conclusion:**

Toxigenic *C. difficile* strains isolated from human CRC mucosal slurries were pro-carcinogenic in mice, suggesting that *C. difficile* is a potential driver of CRC. Given the public health burden of *C. difficile*, further studies are warranted to determine whether *C. difficile* infections (initial, recurrent, and chronic asymptomatic) increase CRC risk in patients.

**Disclosures:**

**Jada Domingue, PhD**, **AstraZeneca** (Employee) **James White, PhD**, **Personal Genome Diagnostics** (Consultant) **Patricia J. Simner, PhD**, **Accelerate Diagnostics** (Grant/Research Support)**Affinity Biosensors** (Grant/Research Support)**BD Diagnostics** (Consultant, Grant/Research Support)**GeneCapture** (Consultant)**OpGen, Inc** (Consultant, Grant/Research Support)**Shionogi, Inc** (Consultant) **Karen C. Carroll, MD**, **MeMed** (Scientific Research Study Investigator)**Meridian Diagnostics, Inc.** (Grant/Research Support)**Pattern Diagnostics** (Advisor or Review Panel member)**Scanogen, Inc.** (Advisor or Review Panel member) **Karen C. Carroll, MD**, Pattern Diagnostics, Inc. (Individual(s) Involved: Self): Grant/Research Support; Scanogen, Inc. (Individual(s) Involved: Self): Consultant **Cynthia L. Sears, MD**, **Bristol Myers Squibb** (Grant/Research Support)**Ferring** (Advisor or Review Panel member)**Janssen** (Grant/Research Support)

